# Supplementary figures and images for: Metabolic Engineering Interventions for Sustainable 2,3-Butanediol Production in Gas-Fermenting Clostridium autoethanogenum
Source: mSystems. 2022 Mar 24;7(2):e01111-21. doi: 10.1128/msystems.01111-21 (PMC9040633; doi:10.1128/msystems.01111-21)

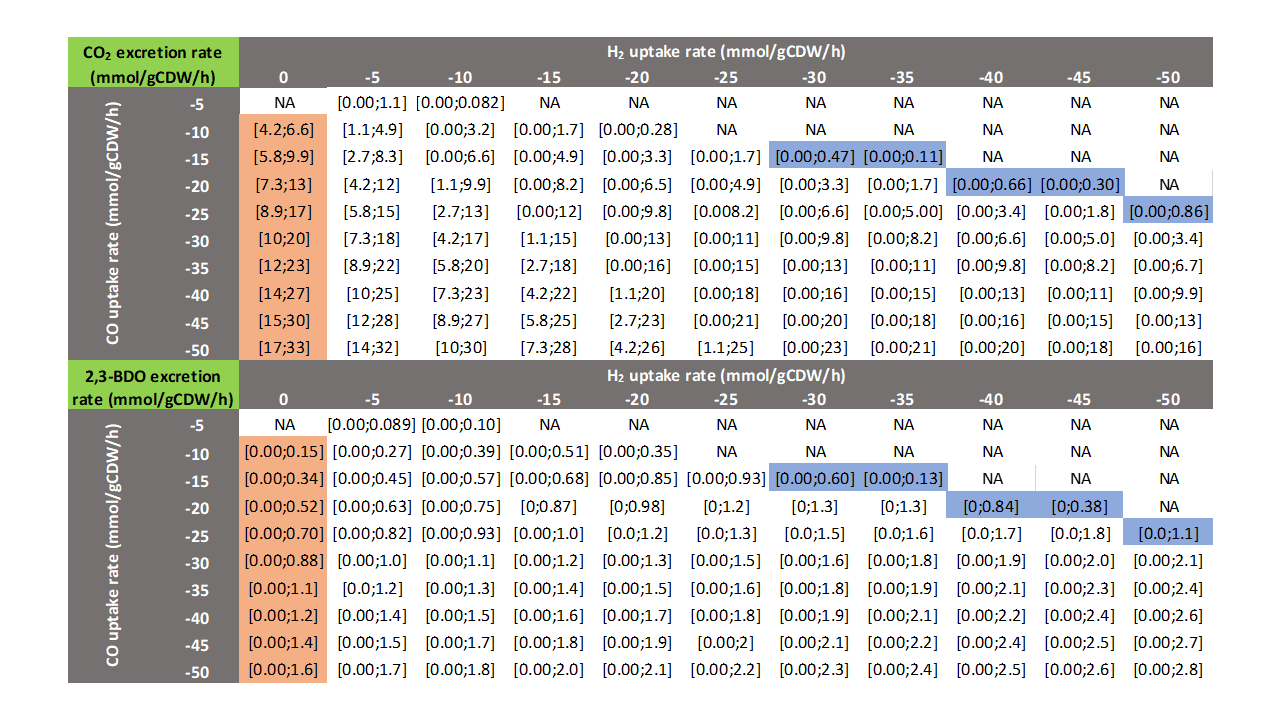

Supplement: FIG S1 [file msystems.01111-21-s0002.tif]

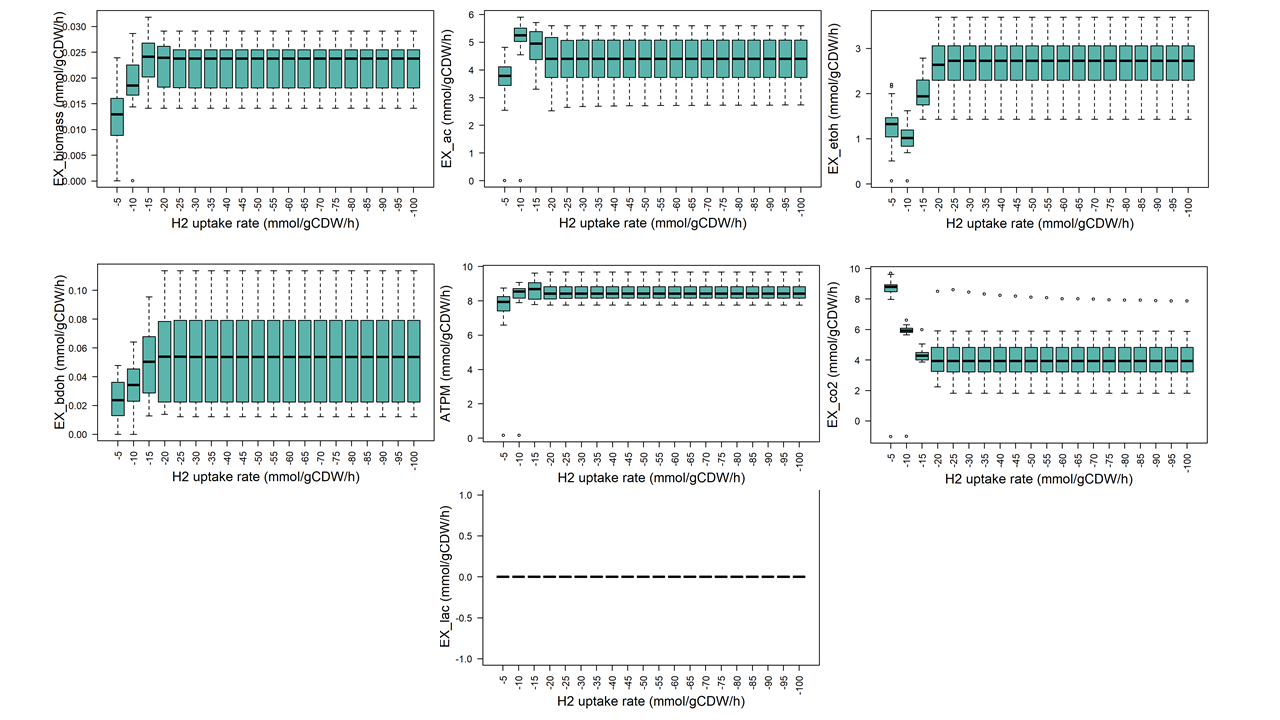

Supplement: FIG S2 [file msystems.01111-21-s0003.tif]

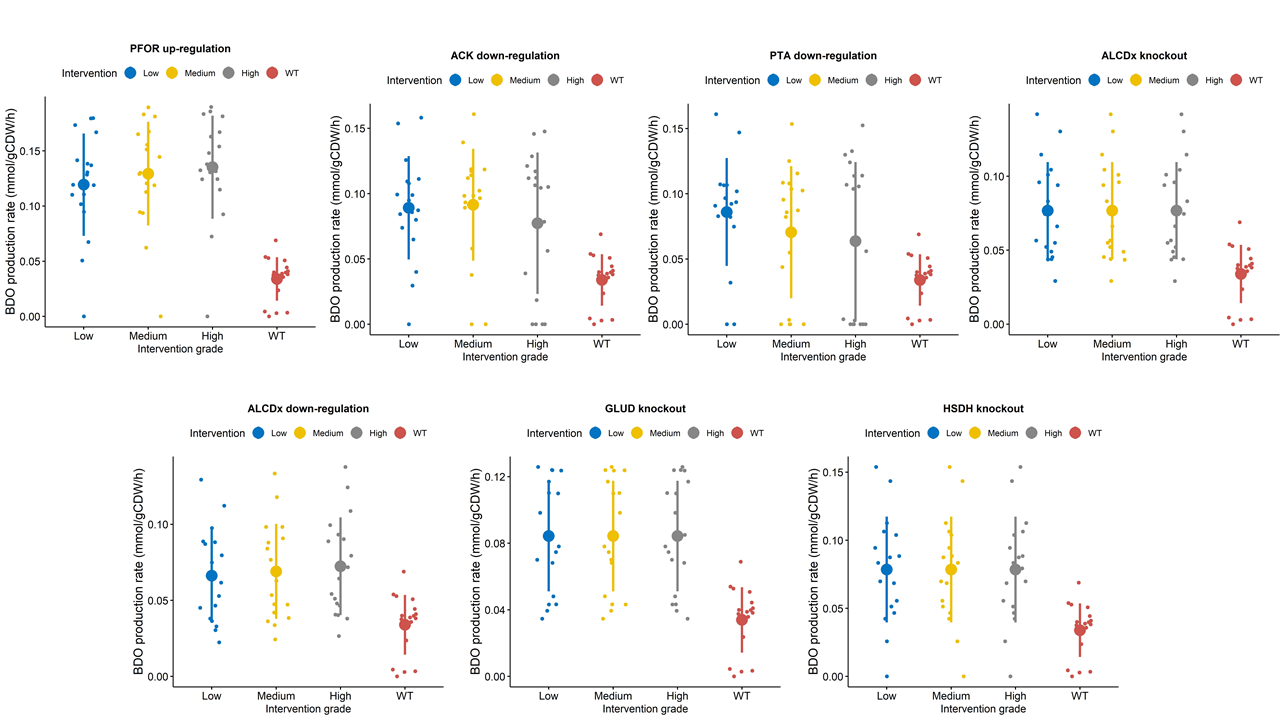

Supplement: FIG S3 [file msystems.01111-21-s0004.tif]

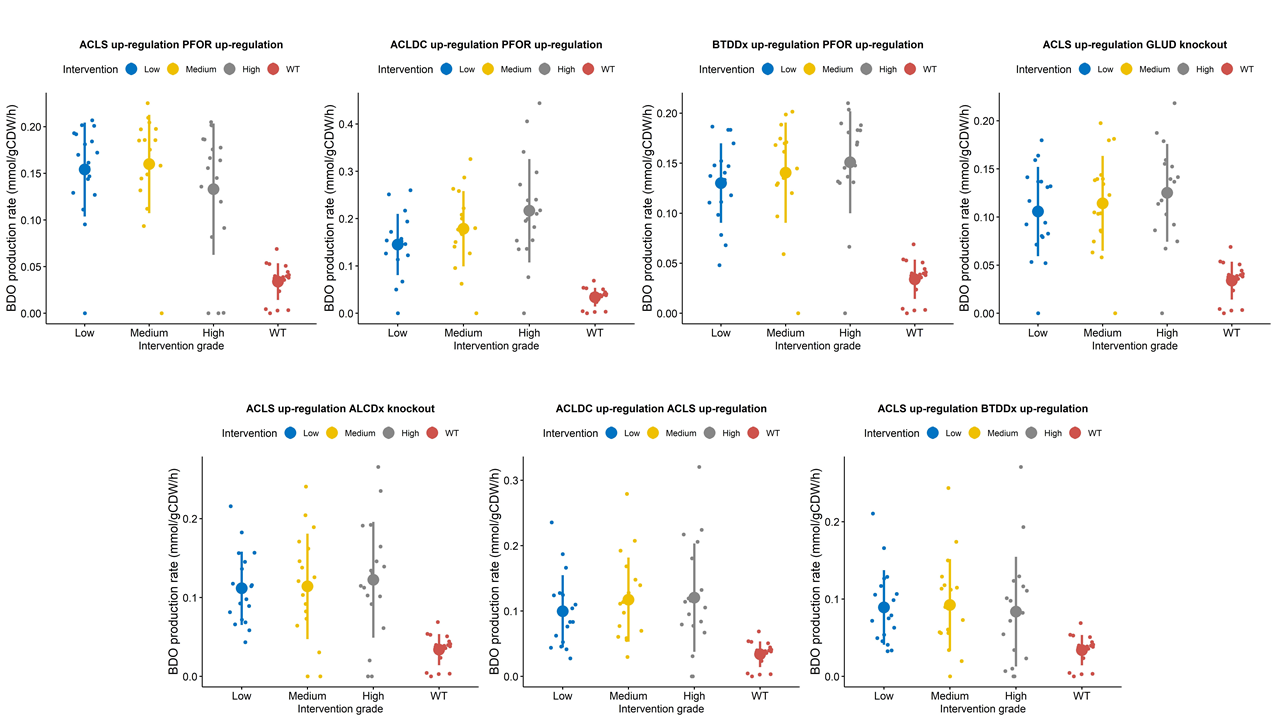

Supplement: FIG S4 [file msystems.01111-21-s0005.tif]

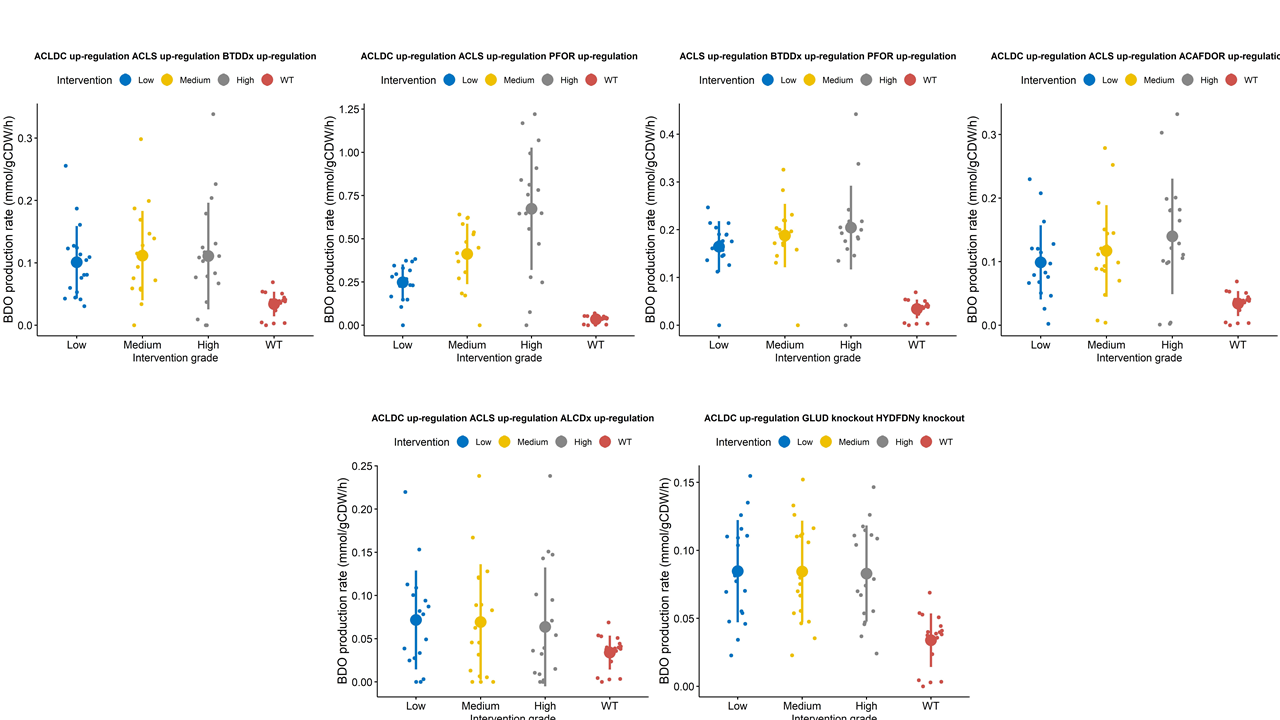

Supplement: FIG S5 [file msystems.01111-21-s0006.tif]
